# Comprehensive Transcriptome Analysis of Response to Nickel Stress in White Birch (*Betula papyrifera*)

**DOI:** 10.1371/journal.pone.0153762

**Published:** 2016-04-15

**Authors:** Gabriel Theriault, Paul Michael, Kabwe Nkongolo

**Affiliations:** 1 Biomolecular Sciences Program, Laurentian University, Sudbury, Ontario, P3E 2C6, Canada; 2 Department of Biology, Laurentian University, Sudbury, Ontario, P3E 2C6, Canada; Friedrich Schiller University, GERMANY

## Abstract

White birch (*Betula papyrifera*) is a dominant tree species of the Boreal Forest. Recent studies have shown that it is fairly resistant to heavy metal contamination, specifically to nickel. Knowledge of regulation of genes associated with metal resistance in higher plants is very sketchy. Availability and annotation of the dwarf birch (*B*. *nana*) enables the use of high throughout sequencing approaches to understanding responses to environmental challenges in other *Betula* species such as *B*. *papyrifera*. The main objectives of this study are to 1) develop and characterize the *B*. *papyrifera* transcriptome, 2) assess gene expression dynamics of *B*. *papyrifera* in response to nickel stress, and 3) describe gene function based on ontology. Nickel resistant and susceptible genotypes were selected and used for transcriptome analysis. A total of 208,058 trinity genes were identified and were assembled to 275,545 total trinity transcripts. The transcripts were mapped to protein sequences and based on best match; we annotated the *B*. *papyrifera* genes and assigned gene ontology. In total, 215,700 transcripts were annotated and were compared to the published *B*. *nana* genome. Overall, a genomic match for 61% transcripts with the reference genome was found. Expression profiles were generated and 62,587 genes were found to be significantly differentially expressed among the nickel resistant, susceptible, and untreated libraries. The main nickel resistance mechanism in *B*. *papyrifera* is a downregulation of genes associated with translation (in ribosome), binding, and transporter activities. Five candidate genes associated to nickel resistance were identified. They include Glutathione S–transferase, thioredoxin family protein, putative transmembrane protein and two Nramp transporters. These genes could be useful for genetic engineering of birch trees.

## Introduction

Nickel is an essential micronutrient for plants with concentrations ranging from 0.01 to 5.00 mg / kg. A low level of bioavailable nickel in soil has a beneficial effect on plant growth, but excess accumulation of this heavy metal in plants causes structural, metabolic, and physiological constraints that affect growth and development [1, 2].

White birch (*Betula papyrifera*) is a dominant tree species of the boreal forest. It is a pioneer species and rapidly colonizes open areas [3]. It is the predominant species in mining regions contaminated with metals in Northern Ontario, Canada (especially in the Greater Sudbury Region), as it represents 60% of all tree species in many areas, [4]. This region is known as one of Canada’s most ecologically disturbed areas. Over 100 million tonnes of sulfur dioxide and tens of thousands of tonnes of cobalt (Co), copper (Cu), nickel (Ni), and iron (Fe) have been released from roast pits and smelters [5]. This caused localized heavy metal pollution and acidification of surrounding ecosystems since industrial activities started over 100 years ago [6]. Little is known about *B*. *papyrifera* adaptation to soil metal contamination even though it plays such a key role in forest sustainability. Recent studies have shown that this species is fairly resistant to heavy metal contaminations specifically to nickel [7, 8].

Cellular mechanisms of nickel tolerance are unknown. In general, metal tolerance includes detoxification processes, complexation by phytochelatins, phylates, amino acids and organic acids, and compartmentation of toxic ions within the cell vacuole and apoplast [2]. It is also known that toxic metal—induced oxidative stress is usually greater in sensitive plants than in tolerant plants, which shows reduced lipid peroxidation. In general, plants are rarely adapted to high concentrations of metals and enzyme activity is usually decreased or lost when they are under metal stress. Studies suggest that glutathione (GSH) and its related metabolizing enzymes, proteins, and peptides play a pivotal role in heavy metal tolerance by controlling reactive oxygen species (ROS) and methylglyoxal (MG) detoxification, heavy metal (HM) uptake, translocation, chelation, and detoxification [9]. Theriault *et al*. [10] showed that *B*. *papyrifera* is a nickel accumulator and it survives despite high concentrations of nickel in its aerial shoots. This is because accumulator plants biotransform contaminants into inert forms in their tissues. Analysis of segregating *B*. *papyrifera* populations exposed to a high dose of nickel in controlled environment suggested that this tolerance to nickel is controlled by a single recessive gene [11]. In general, resistant plants develop specific mechanisms for uptake, translocation and storage of nutrients and toxic elements. It is very likely that these mechanisms are genetically closely regulated. Knowledge of regulation of genes associated with metals in higher plants is unclear. Genome sequence of only one birch species, dwarf birch (*B*. *nana*), has been completed [12]. To date, transcriptome analysis in the genus *Betula* has not been investigated. Comparative transcriptome analysis is a tool for genetic characterization for stress resistance in plant populations [13, 14, 15]. The main objectives of this study are to 1) develop and characterize white birch (*B*. *papyrifera*) transcriptome, 2) assess gene expression dynamics of *B*. *papyrifera* in response to nickel stress, and 3) describe gene function based on ontology.

## Materials and Methods

### Ethics Statement

*Betula papyrifera* (white birch) seed sampling was conducted on the Laurentian University (Sudbury, Ontario) experimental site. Specific permission was not required to access this location.

### Nickel treatments

*Betula papyrifera* seeds collected from the Laurentian University experimental site in the Greater Sudbury Region (Northern Ontario) were stored at 4°C. They were then germinated on wet filter papers in Petawawa boxes at 27°C. Seedlings were transplanted into pots containing a topsoil/peat moss mixture. After a month, they were fertilized with equal amounts of nitrogen, phosphorus and potassium (20-20-20) and left to grow for four additional months at 27°C in a growth chamber. They were then transplanted into a 50:50 mix of quartz sand and peat moss and left to grow for another month. To assess the toxicity of nickel, segregating populations were treated with a single dose of Ni(NO_3_)_2_ salt which led to a final concentration of 1,600 mg /kg of nickel as previously described [11]. This concentration corresponds to the total amount of nickel in contaminated sites in the mining region in Northern Ontario. Water treated plants were used as reference. To determine any effect of nitrate on plant damage, KNO_3_ was also used as an additional control (nitrate control). The experimental design was a completely randomized block with 15 replications.

**Fig 1:**
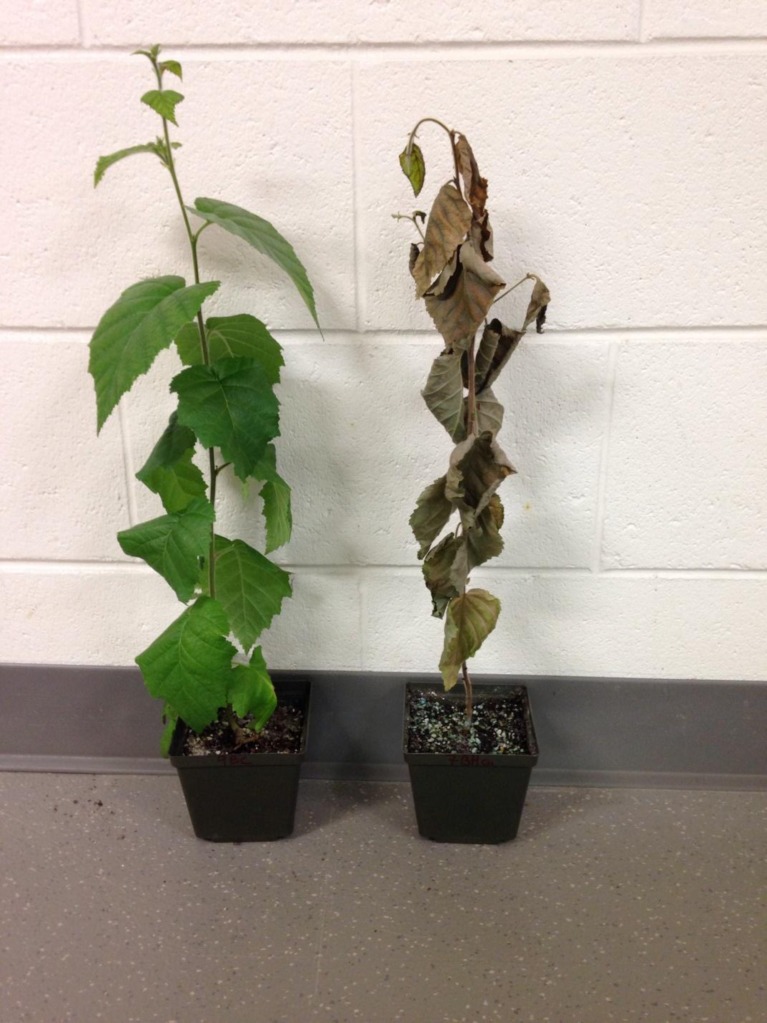
White birch (*Betula papyrifera*) from M2 populations treated with 1,600 mg/kg of nickel (Ni). Left and right plants are resistant (damage rating score of 1) and susceptible (damage rating score of 9) seven days after Ni treatment, respectively.

Damage rating was recorded every two days based on a scale of 1 to 9, 1 = no visible toxicity symptoms and 9 = dead plants. Individual plants with a score of 1 to 3 were considered nickel resistant, 4 to 6, moderately resistant/susceptible, and 7 to 9 susceptible. Fig 1 depicts the damage rating scale range.

### Nickel content analysis

Total nickel in roots and leaves was measured as described in [16] and [17]. The detection of total Ni was determined using inductively coupled plasma atomic emission spectrometry (ICP-AES). The quality control program, completed in an ISO 17025 accredited facility (The Forest Resources & Soil Testing (FoReST) Laboratory, Faculty of Natural Resources Management (Lakehead University Center for Analytical Services (LUCAS), Thunder Bay, Ontario, Canada), included analysis of duplicates, Certified Reference Materials (CRM’s), Internal Reference Materials (IRM’s), procedural and calibration of blanks. Continuous calibration verification and use of internal standards (Sc, Y, Bi) was done to correct for any mass bias. All concentrations were calculated in mass/mass dry soil basis. The data obtained for all elements of interest in analyzed CRM soil samples were within ± 12% of the certified level.

Nickel concentration data were statistically analyzed using SPSS 20 for Windows, with all data being transformed using a log_10_ transformation to achieve a normal distribution. ANOVA, followed by Tukey’s HSD multiple comparison analysis, was performed to determine significant differences (P ≤ 0.05) among Ni content in soil, root, and leaf samples.

### RNA extraction

Total RNA was extracted using the Plant/Fungi Total RNA Purification kit from Norgen Biotek Corporation (Thorold, Canada). It was quantified using the Qubit^(R)^ RNA BR Assay kit provided by Life Technologies (Carlsbad, United States). The quality of the RNA was verified on a 1% agarose gel.

### *De novo* transcripts assembly

**Fig 2:**
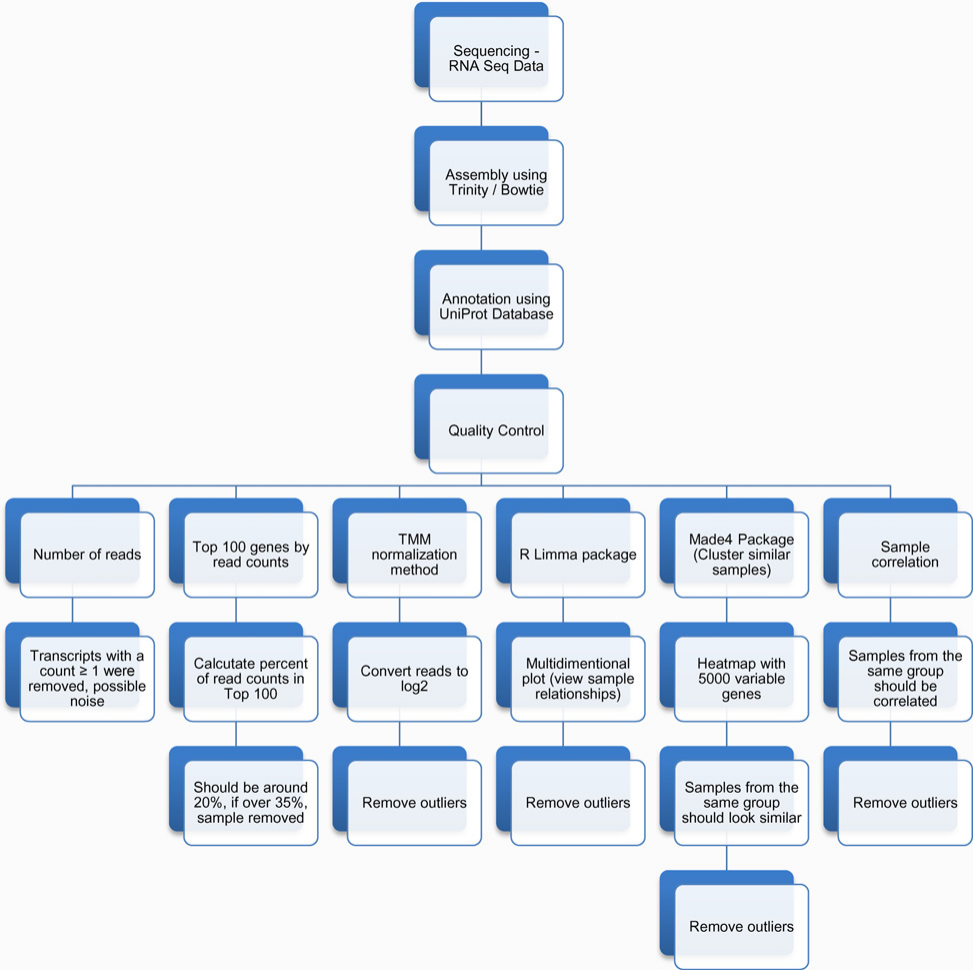
Flowchart of the *Betula papyrifera* transcriptome analysis. Based on the data, we removed three samples as outliers.

RNA- seq libraries were generated using the TruSeq RNA-Seq Sample Prep Kit according to the manufacturer’s protocol (Illumina Inc. San Diego, CA). Poly-A RNA was isolated from total RNA and chemically fragmented. First and second strand cDNA syntheses were followed by end repair and adenosines were added to the 3’ ends. Adapters were ligated to the cDNA and 200 ± 25 bp fragments were gel—purified and enriched by PCR. The library was quantified using Bioanalyzer 2100 (Agilent Technologies, Santa Clara, CA) and the sequencing was performed on the Illumina HiSeq 2000 sequencing system (Illumina Inc.) at Seq Matic (Fremont California, USA). The RNA- sequence data from all the samples including 9 nickel-treated (3 resistant, 3 moderately resistant/susceptible, 3 susceptible), 3 water-treated (control), and 3 nitrate-treated were used as input to the Trinity program (http://trinityrnaseq.githb.io) to assemble the transcripts. A flowchart summarizing all the steps and quality controls leading to candidate genes selection is described in Fig 2. All transcripts were mapped to protein sequences in the UniProt database (http://www.uniprot.org/) and the best match was used to annotate genes and assign gene ontology information.

We counted the number of genes that had at least 1, 2, 10, 50 or 100 counts. In general, the number of genes with two or more counts was used as a rough estimate of how many genes were expressed. Genes with only one read could be noise. In addition, the number of genes with 10 or more reads is a good indicator of how many genes have enough reads for downstream statistical analysis. Basically, the genes were ranked by read counts, and the number of reads belonging to the top genes was computed (up to top 100). If the majority of the reads came from top genes, then the sample probably had bottlenecking issues where a few genes were amplified many times by PCR during library preparation.

### Number and expression of genes detected

The raw reads were mapped to Trinity assembled transcripts using bowtie (http://bowtie-bio.sourceforge.net/index.shtml), and RSEM (http://deweylab.biostat.wisc.edu/rsem) was used to quantify transcript and gene expression levels (both gene counts and FPKM levels). Additional QC at gene level was performed, including number of genes detected, percentage of reads belonging to the top genes, normalization for RNA composition, and grouping, and correlation between samples. Gene expression was calculated and expressed as Reads Per Kilobase per Million reads mapped (RPKM) [18]. The count per million (CPM) cutoff was 0.48 based on the average read count of all samples (20.9 million). This CPM cutoff roughly equals to 10 raw reads in this experiment. A gene with a CPM value > 0.48 in at least two samples from the experiment was included for downstream analysis. The raw counts were normalized using voom (http://genomebiology.com/2014/15/2/R29) method from the R Limma package (http://www.bioconductor.org/packages/release/bioc/html/limma.html). After normalization, most samples looked similar. In general, samples with high or low distribution may be outliers (or have large biological differences). Based on expression changes, further analyses were carried out to detect enriched functions. Gene Set Enrichment Analysis (GSEA) method was performed instead of the traditional gene ontology (GO) enrichment method to identify enriched functional categories. This because GSEA does not rely on an arbitrary cutoff, and it can be more sensitive for small changes that happen across a whole group of genes. In addition, GSEA can be applied even when there is no replicate. Finally, the presence of an expression gradient in the samples was evaluated after baseline correction. Baseline filtering of genes that likely are the effect of nitrate was conducted.

We used the made4 (multivariate analysis of microarrays data using ADE4) program to cluster samples and draw a heatmap based on genes that have variable expression across samples. These variable genes were chosen based on standard deviation (SD) of expression values larger than 30% of the mean expression value. Genes with mean logCPM < 1 were removed if there are > 5,000 variable genes, and then all genes were ranked by SD/Mean to get the top 5,000 genes. Multidimensional plot was created to view sample relationships. This is done using R Limma package (http://www.bioconductor.org/package/release/bioc/html/limma.html).

The annotated sequences were run through the GO-Slim function of the BLAST2GO program to provide a high level summary of functions that include biological process, cellular components, and molecular function.

### Identification of genes associated with nickel resistance

For the identification of genes associated with nickel resistance, a pairwise comparison between resistant / moderately-resistant and resistant / susceptible which showed all the differentially expressed genes (DEGs) was performed. Top 50 genes based on Log2 FC from each pairwise comparison were ranked. Five candidate genes were selected based on the expression in resistant genotypes compared to susceptible, on their function, and their characterization documented in existing literature.

## Results and Discussion

### Tolerance to nickel

**Fig 3:**
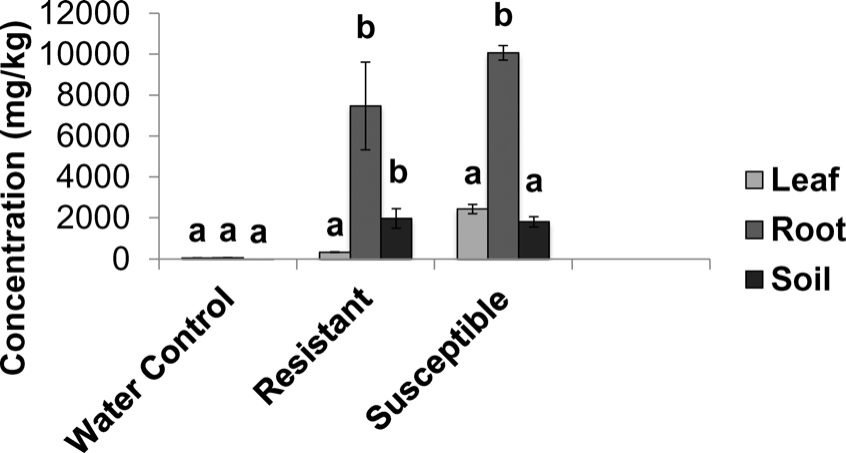
Concentration of nickel in leaves and roots of *Betula papyrifera* growing in soil containing 1,600 mg/kg of nickel. Means with a common subscript are not significantly different based on Tukey multiple comparison test (P ≥ 0.05). Error bars represent standard error.

No damage was observed in water control genotypes (damage rating = 1) and only minor damages were seen in the nitrate control (damage rating = 2). Out of the 15 genotypes tested, five were classified resistant (with damage rating of 1 or 2), 5 moderately susceptible (average damage rating 5.6), and five were susceptible (damage rating of 9). Fig 1 shows a phenotype of a highly resistant and a susceptible plant. Metal analysis revealed that the highly resistant, moderately resistant/susceptible and susceptible plants contain similar levels of nickel in their roots. But a significantly lower amount of nickel was found in leaves of the highly resistant plants compared to moderately resistant and susceptible genotypes (Fig 3). The damage observed in resistant plants remained unchanged for the first four days after the treatment, while the moderately resistant/susceptible showed increasing damage overtime. Metal analysis revealed that resistant, moderately resistant/susceptible and susceptible genotypes showed the same levels of metals in roots, but in resistant plants, nickel translocation from roots to leaves was limited. The levels of Ni in leaves in moderately resistant/susceptible and susceptible genotypes were similar (Fig 3). Hence the moderately resistant/susceptible and the susceptible genotypes were classified as susceptible in this experiment. This was confirmed later by the levels of gene expression based on the transcriptome analysis.

### Transcriptome assembly and analysis

After trimming all the contaminations, a total of 208,058 trinity genes were identified and were assembled to 275,545 total trinity transcripts with an average length of 561.54. This represents a total of 154,728,281 bases. Overall, 215,700 transcripts were annotated and were compared to the published *B*. *nana* genome (http://birchgenome.org). A genomic match for 61% transcripts with the reference genome was found. This Transcriptome Shotgun Assembly project has been deposited at DDBJ/EMBL/GenBank under the accession GEIC00000000. The version described in this paper is the first version, GEIC01000000.

### Gene ontology classification

Transcripts were assigned gene ontology and grouped by biological function, molecular functions, and cellular compartmentation. Overall the numbers of transcripts were similar among the different groups (control, resistant, and susceptible).

**Fig 4:**
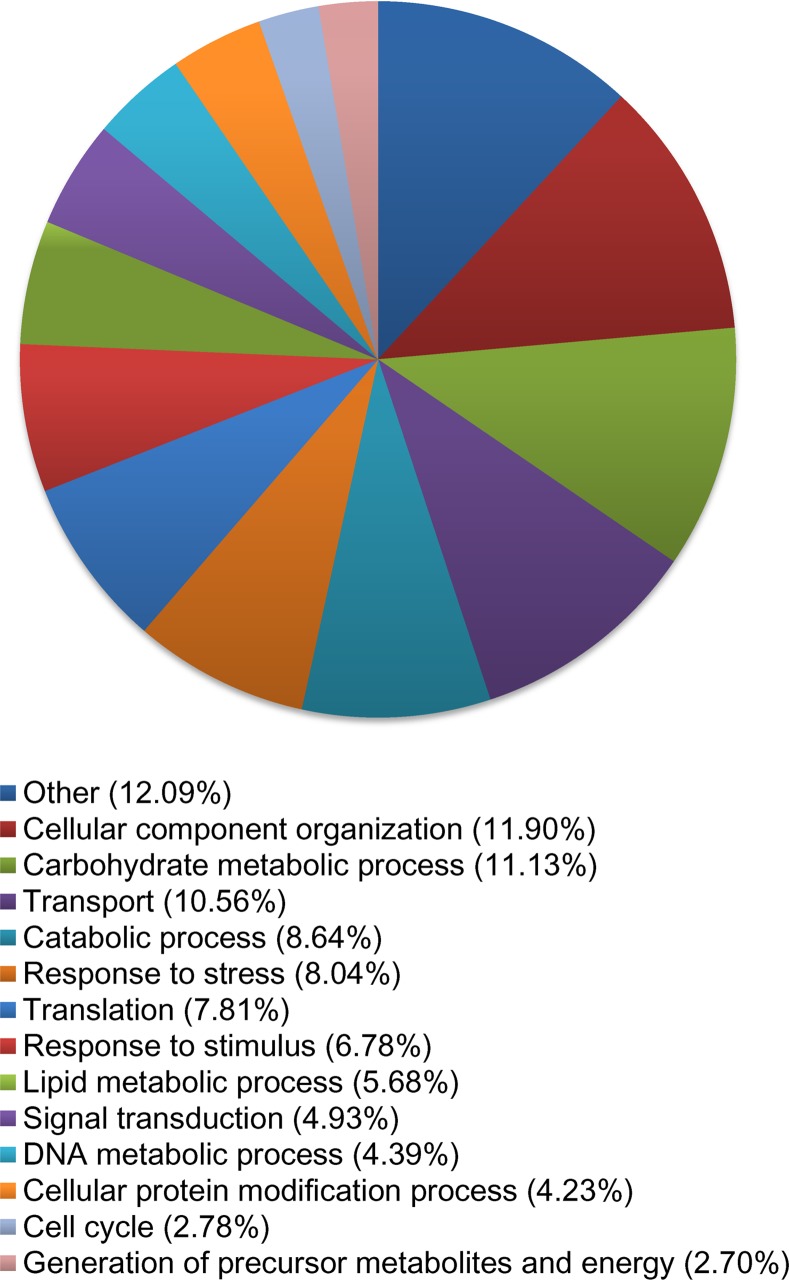
Percentage of transcripts in *Betula papyrifera* control samples. A total of 21,793 transcripts were assigned gene ontology and grouped by biological function using BLAST2GO. Categories under 2% were grouped together and classified as “other”.

**Fig 5:**
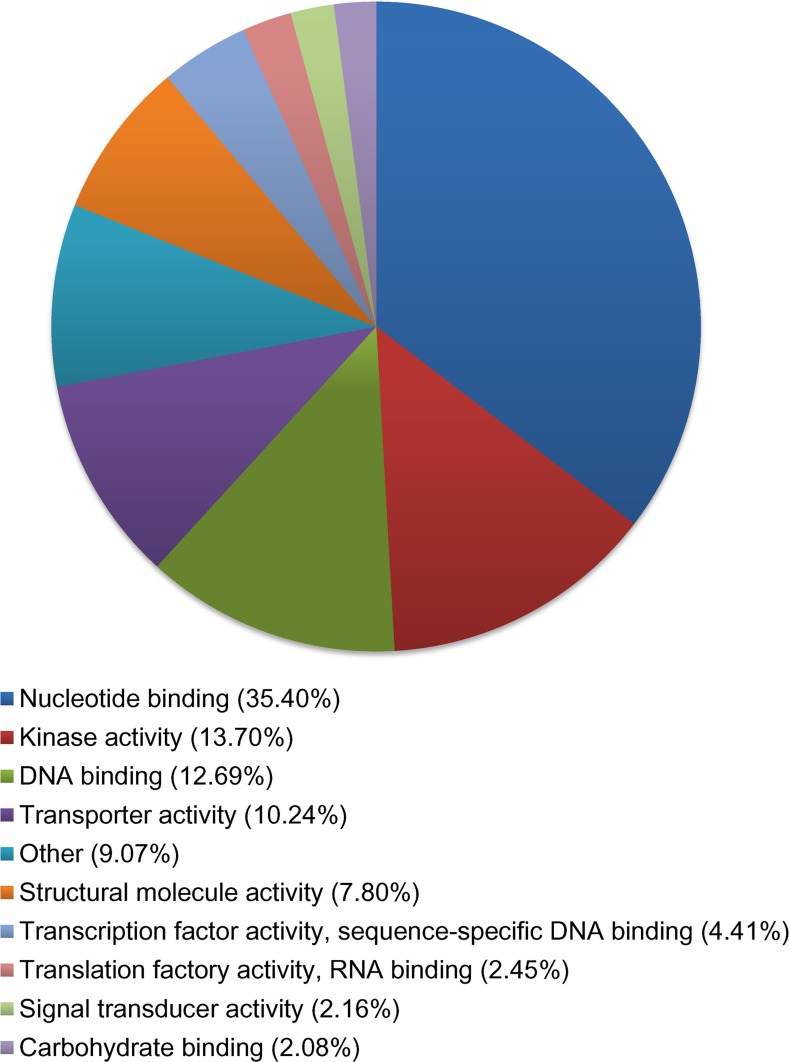
Percentage of transcripts in *Betula papyrifera* control samples. A total of 23,876 transcripts were assigned gene ontology and grouped by molecular function using BLAST2GO. Categories under 2% were grouped together and classified as “other”.

For biological function, 23,876 transcripts were assigned ontology. Roughly, 65% of all categories fall under cellular component organization (CCO), carbohydrate metabolic process (CMP), transport, catabolic process, response to stress, translation, and response to stimulus (Fig 4). For molecular functions, 35% transcripts code for proteins involved in binding activities, 13.7% for kinase activities, 12.7% for DNA binding, and 10.2% for transport activities (Fig 5).

**Fig 6:**
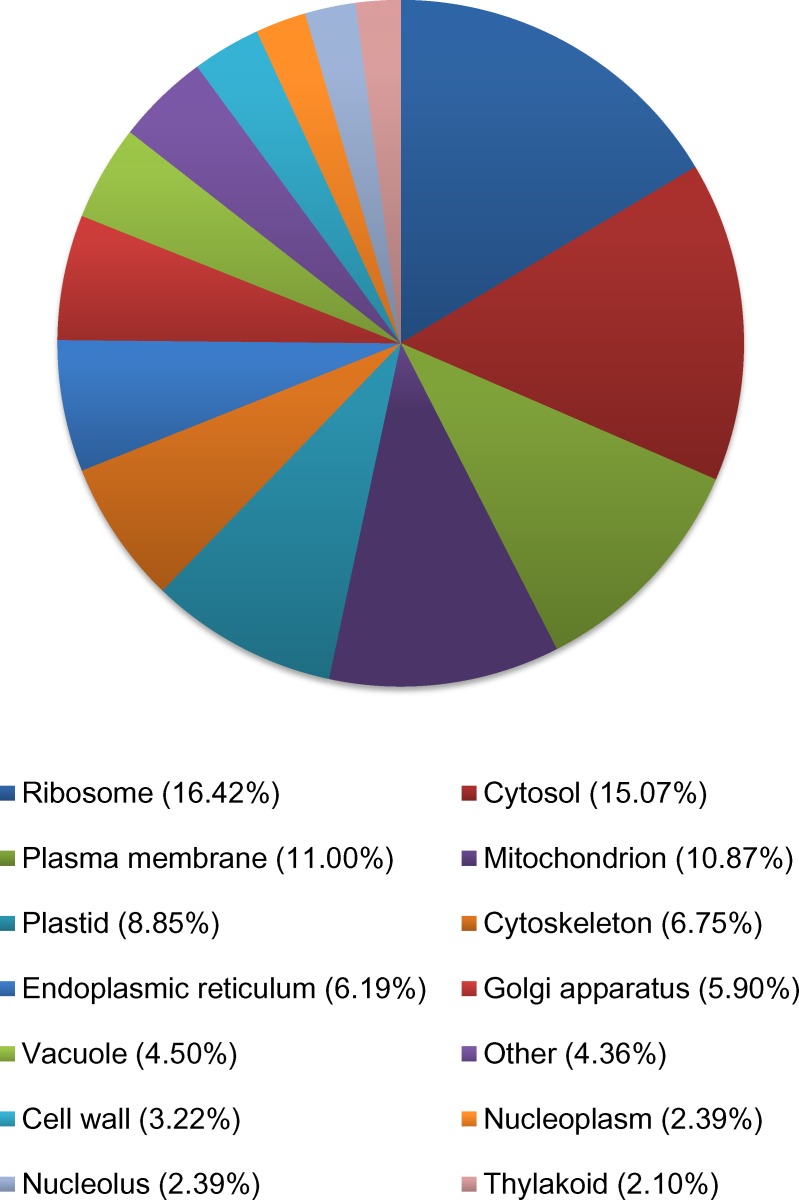
Percentage of transcripts in *Betula papyrifera* from the control. A total of 10,580 transcripts were assigned gene ontology and grouped by cellular compartment using BLAST2GO. Categories under 2% were grouped together and classified as “other”.

For cellular compartmentation, 10,580 transcripts were assigned gene ontology. Overall, 16.4% of the transcripts were localized in ribosome, 15.1% in cytosol, 11% in plasma membrane, 11% in mitochondria, 9% in plastid, 6.8% in cytoskeleton, 6.2% in endoplasmic reticulum, and 5.9% in Golgi apparatus (Fig 6). Hence, among the three principal gene ontologies, most of the differentially expressed genes were classified into the terms CCO, CMP, transport, Nucleotide binding, kinase activities, DNA binding, ribosome, cytosol, plasma membrane, and mitochondria suggesting that these functional processes play a major role in *B*. *papyrifera* gene activities.

### Differential gene expression

**Fig 7:**
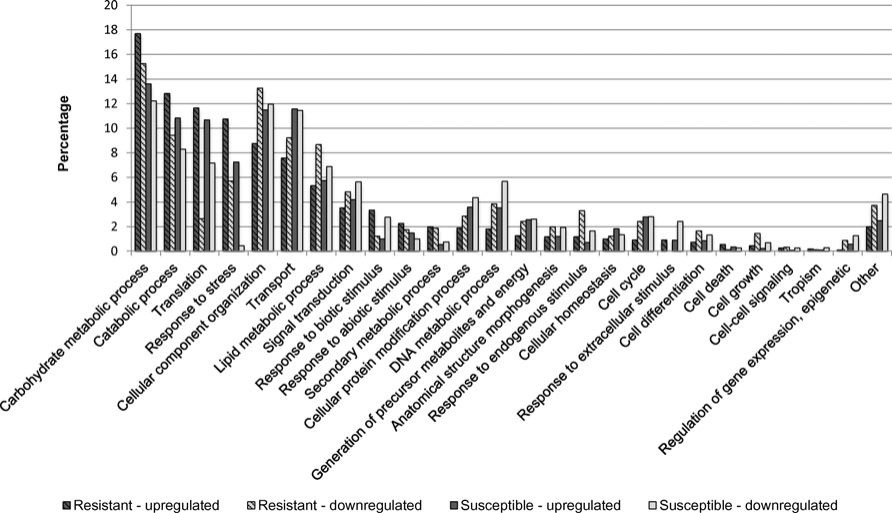
Percentage of upregulated and downregulated transcripts in the nickel—resistant and susceptible *Betula papyrifera* genotypes compared to control (nickel untreated samples). Transcripts were assigned gene ontology and grouped by biological function using BLAST2GO.

**Fig 8:**
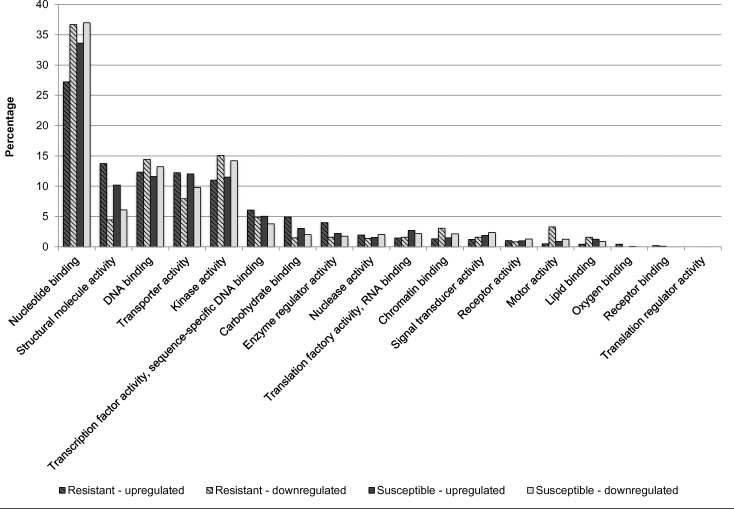
Percentage of upregulated and downregulated transcripts in the nickel—resistant and susceptible *Betula papyrifera* genotypes compared to control (nickel untreated samples). Transcripts were assigned gene ontology and grouped by molecular function using BLAST2GO.

**Fig 9:**
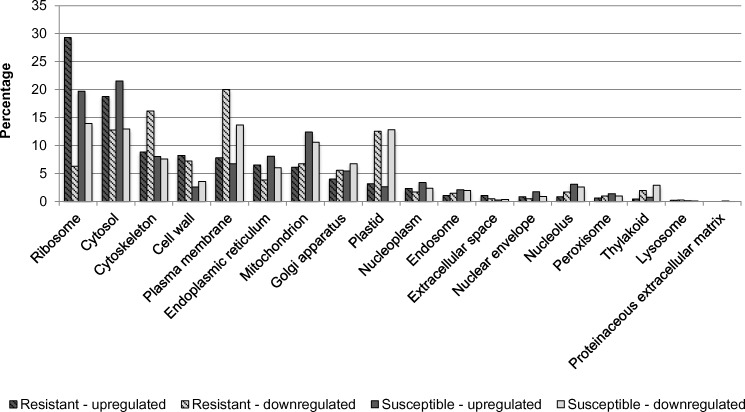
Percentage of upregulated and downregulated transcripts in the nickel- resistant and susceptible *Betula papyrifera* genotypes compared to control (nickel untreated samples). Transcripts were assigned gene ontology and grouped by cellular compartment using BLAST2GO.

Overall, 99,243 of the total 208,058 genes were expressed. After normalization, a total of 62,587 genes were selected as effectively expressed. Classification of differentially expressed genes was performed to examine their functional distribution characteristics. To determine the effect of nickel treatment, the whole transcriptome was analyzed and nickel–resistant and susceptible were compared to untreated samples. There was a higher number of upregulated than downregulated translation transcripts for biological functions. The same trend was observed for response to stress genes (Fig 7). Analysis of molecular functions reveals a high level of upregulation of structural molecule activities for resistant and susceptible genotypes compared to control (Fig 8). For cellular compartment, there was an upregulation of ribosome genes in both resistant and susceptible, with a higher increment of regulation in resistant samples. A down regulation of cytoskeleton and plasma membranes genes was observed in resistant genotypes (RG) and in plasma membrane genes in susceptible genotypes (SG). Plastid genes were downregulated in both RG and SG (Fig 9).

### Pairwise comparison of resistant and susceptible genotypes

When highly resistant genotypes were compared to control, 3,225 genes were upregulated and 3,012 downregulated while 6,415 were upregulated and 2444 downregulated when susceptible genotypes were compared to control. On the other hand, we found 1,646 upregulated and 1,124 downregulated genes when susceptible genotypes were compared to resistant. There were no differences in gene expression when moderately resistant/susceptible and susceptible genotypes were compared. This confirmed that the moderately resistant/susceptible genotypes are susceptible genotype with a delayed expression of damage when treated with a high dose of nickel.

**Fig 10:**
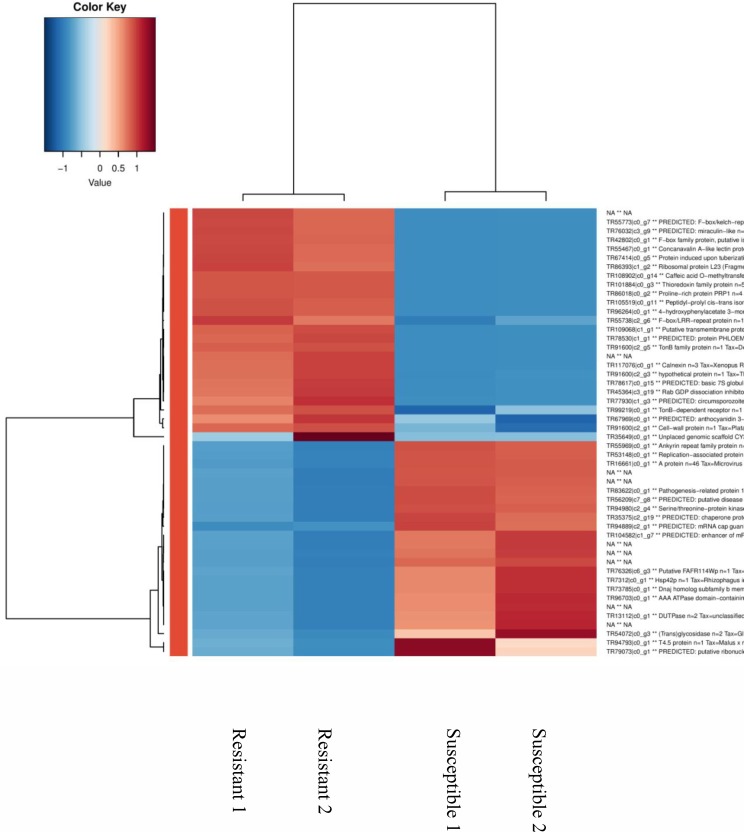
Top 50 differentially expressed genes between nickel-treated resistant and susceptible *Betula papyrifera* genotypes based on logFC. The red colour represents an upregulation and blue downregulation.

**Fig 11:**
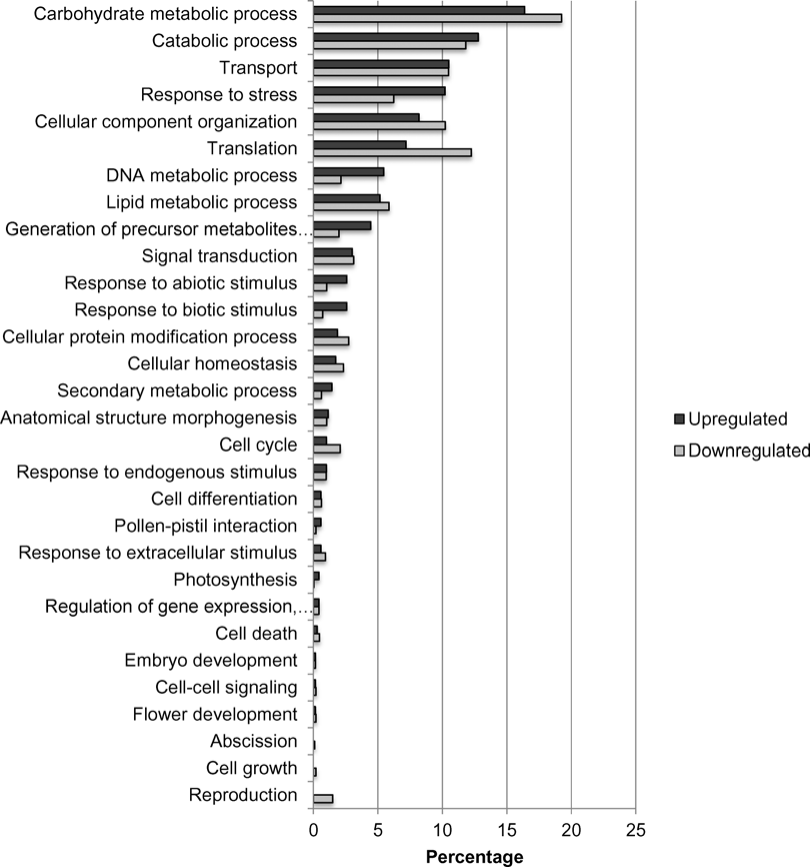
Percentage of upregulated (total number = 696) and downregulated (total number = 1,927) transcripts when nickel resistant *Betula papyrifera* genotypes were compared to nickel susceptible genotypes. Transcripts were assigned gene ontology and grouped by biological function using BLAST2GO.

**Fig 12:**
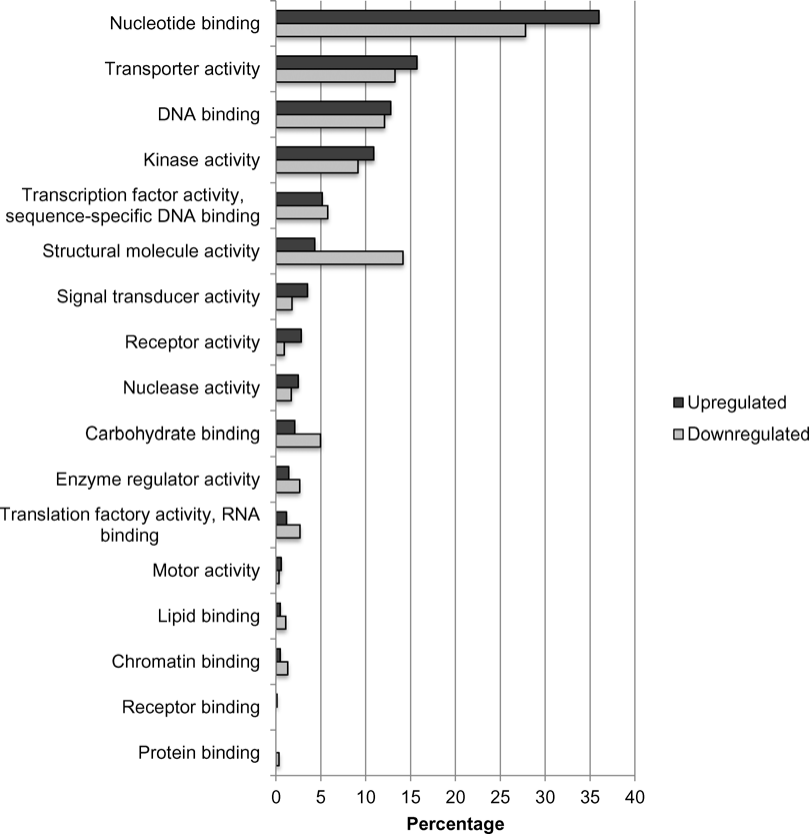
Percentage of upregulated (total number = 853) and downregulated (total number = 1,751) transcripts when nickel resistant Betula *papyrifera* genotypes were compared to nickel susceptible genotypes. Transcripts were assigned gene ontology and grouped by molecular function using BLAST2GO.

**Fig 13:**
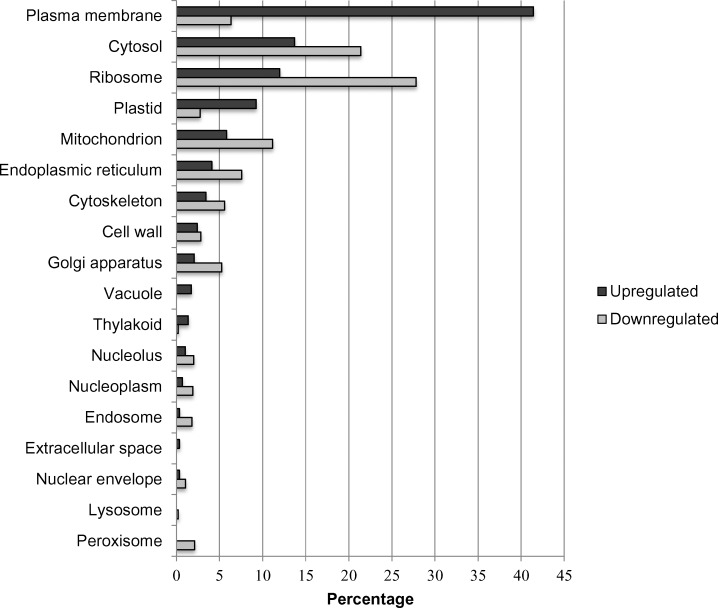
Percentage of upregulated (total number = 292) and downregulated (total number = 949) transcripts when nickel resistant Betula *papyrifera* genotypes were compared to nickel susceptible genotypes. Transcripts were assigned gene ontology and grouped by cellular compartment using BLAST2GO.

Fig 10 shows regulation of the top 50 genes when resistant genotypes (RG) were compared to susceptible; S1 Fig when RGs were compared to control; and S2 Fig when SGs were compared to control. Pairwise comparison for biological, molecular functions and cellular compartments between RG and SG are described in Figs 11–13. For biological functions, there were more upregulated than downregulated genes in RG compared to SG for response to stress, DNA metabolic process, generation of precursor metabolites, and response to biotic and abiotic stimuli (Fig 11). The opposite trend (more downregulated than upregulated genes) was observed for carbohydrate metabolite process, cellular component organization, translation, and cell cycle. For molecular function, the number of upregulated genes was higher than downregulated for nucleotide binding, signal transducer and receptor activities. The opposite was observed for structural molecular activity and carbohydrate binding (Fig 12). For cellular compartment, we observed 7x more upregulated genes than downregulated in plasma membrane but 2x more downregulated than upregulated in ribosomes, and 60% more downregulated genes than upregulated in cytosol (Fig 13). Comparative analysis of RGs and control, and SGs and control are described in S3–S8 Figs.

### Characterization of highly expressed genes

**Fig 14:**
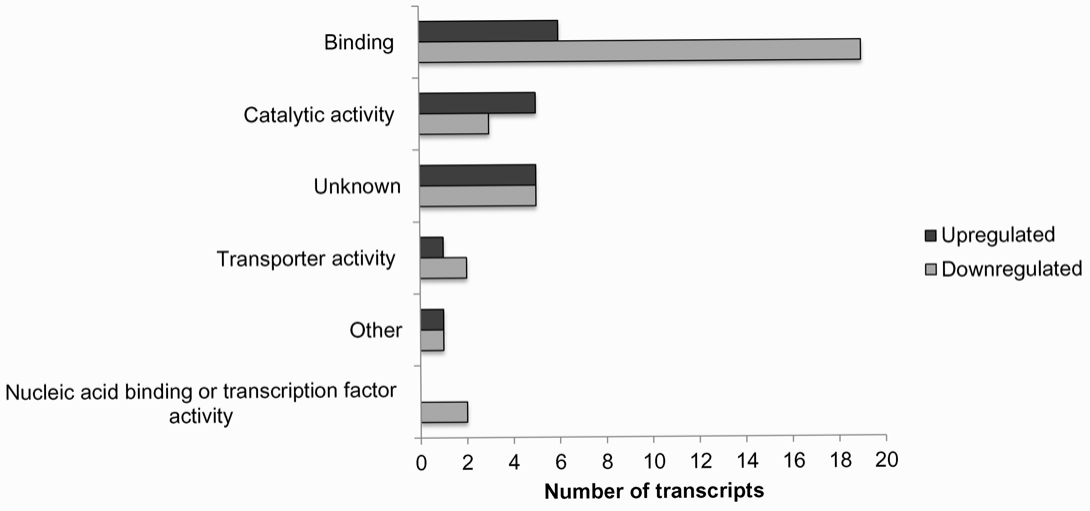
Top 50 transcripts when nickel resistant Betula *papyrifera* genotypes were compared to control (nickel untreated genotypes). Transcripts were assigned gene ontology and grouped by molecular function using BLAST2GO.

Molecular function data shows that among the top 50 transcripts/genes, 50% are involved in binding activity, 16% in catalytic activity, 6% in transport activity, 4% in nucleic acid binding transcription factor activity, 4% in other activities, and 20% unknown activities when RG were compared to control (Fig 14). Overall, 80% of the genes involved in binding activity were downregulated and 20% upregulated in nickel treated-resistant genotypes compared to nickel–untreated genotypes. The same trend was observed for genes involved in transporter activity since two of the three genes identified were upregulated in resistant genotypes. An opposite trend was observed for catalytic activity where 6 of the 10 genes were upregulated in resistant genotypes and 4 downregulated (Fig 14).

The specific genes that were upregulated in resistant genotypes include DNA, actin, calcium, and receptor binding; methyl transferase, isomerase/reduction, aconitate hydratase, cellulose synthase, and storage protein genes. The downregulated genes are involved in DNA, metal, GTPase, receptor, cellulose synthase/metal, and lipid bindings; and mRNA, hydrolase / reduction, reduction, storage protein, transporter, pectate lyase, metal binding reduction, cellulose synthase/metal binding, DNA binding nuclease, and DNA binding peptidase.

**Fig 15:**
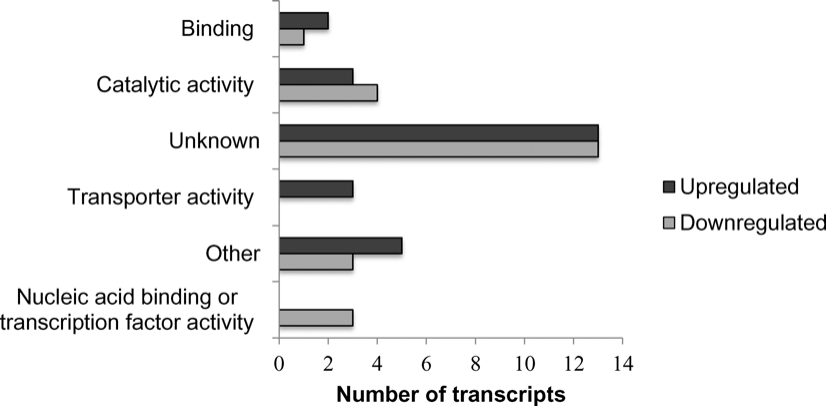
Top 50 transcripts when nickel resistant Betula *papyrifera* genotypes were compared to nickel susceptible genotype. Transcripts were assigned gene ontology and grouped by molecular function using BLAST2GO.

We found that 50% of the top 50 transcripts have unknown molecular functions based on gene ontology when RG and SG were compared. All the transport activity genes were upregulated in RG and all the genes for nucleic binding or transcription factor activity were downregulated (Fig 15). We discovered five genes that were expressed at significantly higher levels in RG compared to SG (from 300 to 500 fold). We further characterized these highly expressed transcripts in RG by querying against NCBI’s ‘nr” database and assigned GO terms in Blast2GO. These candidate genes involved in nickel resistance include Glutathione S–transferase (GST), thioredoxin family protein, putative transmembrane protein and Nramp transporters (Table 1). The expression of these genes was confirmed by q-PCR using designed primers described in Table 2.

**Table 1:** Candidate genes involved in nickel resistance of white birch (*Betula papyrifera*).

Transcript ID	Protein	Log2FC	Function	References
TR73973|c3_g5	Glutathione S-transferase	9.15	Detoxification of electrophilic xenobiotics using glutathione	[24], [23]
TR101884|c0_g3	Thioredoxin family protein	9.15	Defense against heavy metal stress, metal binding	[37], [38]
TR109068|c1_g1	Putative transmembrane protein	9.10	Unknown	None
TR56135|c0_g6	Nramp transporter	8.62	Metal transport, found to play a role in Ni resistance and homeostasis in plants.	[39], [27]
TR56135|c0_g1	Nramp transporter	8.32	Metal transport, found to play a role in Ni resistance and homeostasis in plants.	[39], [27]

Note: Log2FC is calculated based on susceptible white birch

**Table 2:** Primer used for real-time quantitative RT-qPCR verification.

Target gene	Forward primer (5’-3’)	Reverse primer (5’-3’)	**Product size (bp)**
Glutathione S-transferase	TCTGAAACTCAAGGGTGTTGATT	GACTGGAACCTTTTTGTGAACTG	100
Thioredoxin family protein	GAAAAGCTTCTTCAGATCTGGGT	GACTTGGCCTTTCTAAAACTTGC	103
Putative transmembrane protein	TTCTAATAAGGTATTGTGCGCGT	GGAGGAAAAGATTCACCAAGAGT	221
Nramp transporter1	TACATTCTCGCCGTCATTTATCT	GTTGATGCCTTTGATCTTGAAAC	205
Nramp transporter2	CTAGCAAGATCAGAGAGATGGGA	GAAACTTTCTCCATCCTGGTTTC	201

GSTs are a superfamily of multifunctional enzymes that play a role in enzymatic detoxification of xenobiotics. Many GSTs can also act as glutathione peroxidases to scavenge toxic peroxides from cells. In plants, GSTs also provide protection against oxidative stress induced by abiotic stresses and oxidants [19, 20, 9]. An increase in GST activity in both root and shoot tissues was observed in response to Ni stress in wheat (*Triticum aestivum*) and cadmium stress in *Aradopsis thaliana* [21, 22]. Freeman *et al*. [23] showed that elevated glutathione (GSH) concentrations are involved in conferring tolerance to Ni-induced oxidative stress in *Thlaspi spp*., Ni hyperaccumulators. The role of GSH in metal homeostasis, antioxidative defense, and signal transduction under metal stress is discussed in details in [24].

Thioredoxins play a role in oxidative damage avoidance by supplying reducing power to reductases detoxifying lipid hydroperoxides or repairing oxidized proteins. They could act as regulators of scavenging mechanisms and as components of signaling pathways in the plant antioxidant network [25]. Thioredoxin (Trx), peroxiredoxin (Prx), and sulfiredoxin (Srx) in plant cells have been involved in the control of dithiol–disulfide exchanges of target proteins, which modulate redox signaling during plant adaptation to stress.

Nramp (Natural resistance-associated macrophage protein) defines a novel family of related proteins, which have been implicated in the transport of divalent metal ions. This gene family has been highly conserved during evolution and homologues have been found in a wide range of living organisms including bacteria, yeast, insects, mammals and also higher plant [26]. Analyses of the expression of Nramp genes in *Arabidopsis* suggest that all these Nramp genes play roles in constitutive metal transport mostly iron and cadmium. Mizuno *et al*. [27] elucidated the role of Nramp metal-transporters for Ni^2+^- transport and homeostasis. They found that, the expression of TjNramp4 caused elevation of Ni^2+^ sensitivity and Ni^2+^concentrations in yeast. The present study is the first to associate Nramp genes expression with both resistance to and accumulation of nickel. A putative transmembrane protein (TMP) associated with nickel resistance was also identified. The role of this TMP in metals transport in plants has not been investigated.

Recent studies have shown a strong association between heavy metal tolerance and antioxidative and glyoxalase systems, in which plants with low antioxidant capacity exhibit susceptibility to HM toxicity [28, 23, 9]. An increase of at least glutathione (GSH) biosynthesis plays an important role in nickel tolerance in *Thlaspi spp* [9]. Moreover, in several plants, the Ni-induced changes in activity of reactive oxygen species (ROS)-scavenging enzymes, including superoxide dismutase (SOD), peroxidase (POD), and catalase (CAT) were detected [29, 30, 31]. Plants usually respond to oxidative stress by elevating the activity of the antioxidant enzymes of the ascorbate-glutathione cycle such as catalase, peroxidase, superoxide dismutase, glutathione reductase, and ascorbate oxidase, which protect plant cells against free radicals [32, 29, 33]. These activities appear to be reduced in Ni-accumulators plants to which *B*. *papyrifera* belongs [29]. It is not clear if the changes in enzyme activities are triggered directly by Ni^2+^ effects including binding to SH-group or histidine or displacing the metals from metal-enzyme active centers, or indirectly when mediated by chain of reactions that affect the expression of the corresponding genes or exhaust their substrate pools [33].

The role of heavy metal transporters has been investigated in many studies. However our knowledge of the transport processes for HM across plant membranes at the molecular levels is still very limited [26, 34, 35, 36, 9, 14]. The present study showed a significantly upregulation of genes in ribosomes in samples treated with nickel compared to control. This was consistent with biological function profile that reveals a significant upregulation of genes associated with translation in nickel treated samples compared to control. When RG were compared to SG, the main nickel resistance mechanism revealed by the gene regulation analysis is a downregulation of genes associated with translation in ribosome. Significant downregulation of genes associated to binding and transporter activity is also a key characteristic of resistance to nickel in *B*. *papyrifera*. Three of the five candidate genes (putative transmembrane protein, Nramp transporters) identified are related to metal transport in plants.

## Conclusion

In the present study, *B*. *papyrifera* transcriptome was developed. A total of 208,058 trinity genes were identified that were assembled to 275,545 total trinity transcripts. Expression profiles were generated and 62,587 genes were found to be significantly differentially expressed among the nickel resistant, susceptible, and untreated libraries. The main nickel resistance mechanism in *B*. *papyrifera* is a down regulation of genes associated with translation (in ribosome), binding, and transporter activities. Five candidate genes associated to nickel resistance were identified. They include Glutathione S–transferase (GST), thioredoxin family protein, putative transmembrane protein, Nramp transporters. These genes can be useful for genetic engineering of birch trees. The results of the present study also demonstrate how next-generation sequencing technologies can be used to access the transcriptome of higher accumulator plants to identify the underlying molecular mechanisms. Future work can be done to use the genomic sequence to improve the transcriptome (e.g. obtain exon/intron structure, merge fragments to get full length genes, identify promoter sequences etc…)

## Supporting Information

S1 Fig Top 50 differentially expressed genes between nickel–treated *Betula papyrifera* resistant genotypes and control (nickel-untreated) based on logFC. The red colour represents an upregulation and blue downregulation.
(TIF)

S2 Fig Top 50 differentially expressed genes between nickel–treated *Betula papyrifera* susceptible genotypes and control (nickel-untreated) based on logFC. The red colour represents an upregulation and blue downregulation.
(TIF)

S3 Fig Percentage of upregulated (total number = 1,108) and downregulated (total number = 912) transcripts when nickel–resistant *Betula papyrifera* genotypes were compared to control (nickel–untreated). Transcripts were assigned gene ontology and grouped by biological function using BLAST2GO.
(TIF)

S4 Fig Percentage of upregulated (total number = 974) and downregulated (total number = 943) when nickel–resistant *Betula papyrifera* genotypes were compared to control (nickel–untreated). Transcripts were assigned gene ontology and grouped by molecular function using BLAST2GO.
(TIF)

S5 Fig Percentage of upregulated (total number = 475) and downregulated (total number = 415) transcripts when nickel–resistant *Betula papyrifera* genotypes were compared to control (nickel–untreated). Transcripts were assigned gene ontology and grouped by cellular compartment using BLAST2GO.
(TIF)

S6 Fig Percentage of upregulated (total number = 15,380) and downregulated (total number = 11,032) transcripts when nickel–susceptible *Betula papyrifera* genotypes were compared to control (nickel–untreated). Transcripts were assigned gene ontology and grouped by biological function using BLAST2GO.
(TIF)

S7 Fig Percentage of upregulated (total number = 16,652) and downregulated (total number = 13,480) when nickel–susceptible *Betula papyrifera* genotypes were compared to control (nickel–untreated). Transcripts were assigned gene ontology and grouped by molecular function using BLAST2GO.
(TIF)

S8 Fig Percentage of upregulated (total number = 8,718) and downregulated (total number = 5,472) transcripts when nickel–susceptible *Betula papyrifera* genotypes were compared to control (nickel–untreated). Transcripts were assigned gene ontology and grouped by cellular compartment using BLAST2GO.
(TIF)

## References

[1] Van Asshe F, Glijster H. Effects of metals on enzymes activity in plant. Plant, Cell Environ. 1990; 13: 195–206.

[2] Molas J. Comparison of nickel toxicity and resistance strategies of cabbage plants grown in soil with addition of inorganic and organic Ni (II) complexes. Dev Plant Soil Sci. 2001; 92: 464–465.

[3] Perala DA, Alm AA. Reproductive ecology of birch: a review. Forest Ecol Manag. 1990; 32(1): 1–38.

[4] Theriault G, Nkongolo KK, Narendrula R, Beckett P. Molecular and ecological characterisation of plant populations from limed and metal-contaminated sites in Northern Ontario (Canada): ISSR analysis of white birch (*Betula papyrifera*) populations. Chem Ecol. 2013; 29(7): 1–13.

[5] Freedman B, Hutchinson TC. Long-term effects of smelter pollution at Sudbury Ontario Canada on forest community composition. Can J Bot. 1980; 58(19): 2123–2140.

[6] Winterhalder K. Environmental degradation and rehabilitation of the landscape around Sudbury, a major mining and smelting area. Environ Rev. 1996; 4: 185–224.

[7] Amiro BD, Courtin GM. Patterns of Vegetation in the Vicinity of an Industrially Disturbed Ecosystem Sudbury Ontario Canada. Can J Bot, 1981; 59(9): 1623–1639.

[8] Kirkey FM, Matthews J, Ryser P. Metal resistance in populations of red maple (Acer rubrum L.) and white birch (*Betula papyrifera* Marsh.) from a metal-contaminated region and neighbouring non-contaminated regions. Environ Pollut, 2012; 164: 53–58. 10.1016/j.envpol.2012.01.012 22336730

[9] Hossain MA, Piyatida P, da Silva JAT, Fujita M. Molecular Mechanism of Heavy Metal Toxicity and Tolerance in Plants: Central Role of Glutathione in Detoxification of Reactive Oxygen Species and Methylglyoxal and in Heavy Metal Chelation. J Bot. 2012 10.1155/2012/872875

[10] Theriault G, Nkongolo KK. Genetic and metal analyses of fragmented populations of *Betula papyrifera* (Marsh) in a mining reclaimed region: identification of population–diagnostic molecular marker. Ecol Evol. 2014; 4(17): 3435–3443. 10.1002/ece3.1195 25535559 PMC4228617

[11] Theriault G, Nkongolo KK. Analysis of nickel and copper toxicity in white birch (*Betula papyrifera*) populations from a mining region: Association with whole-genome DNA methylation. 2015. Proceedings of Sudbury Mining and Environment International Conference, 6 2015.

[12] Wang N, Thomson M, Bodles WJA, Crawford RMM, Hunt HV, Featherstone AW, et al Genome sequence of dwarf birch (*Betula nana*) and cross-species RAD marker. Mol Ecol. 2013; 22: 3098–3111. 10.1111/mec.12131 23167599

[13] Halimaa P, Blande D, Aarts MGM, Tuomainen M, Tervahauta A, Kärenlampi S. Comparative transcriptome analysis of the metal hyperaccumulator *Noccaea caerulescens*. Front Plant Sci. 2014; 5: 213 10.3389/fpls.2014.00213 PMC4033236 24904610

[14] Merlot S, Hannibal L, Martins S, Laetitia M., Amir H, Lebrun M, et al The metal transporter PgIREG1 from the hyperaccumulator *Psychotria gabriellae* is a candidate gene for nickel tolerance and accumulation. J Exp Bot. 2014; 65(6):1551–64. 10.1093/jxb/eru025 24510940

[15] Barghini E, Cossu RM, Cavallini A, Giordani T. Transcriptome analysis of response to drought in poplar interspecific hybrids. Genomics Data 3 143–145. 10.1016/j.gdata.2015.01.004 26484164 PMC4535942

[16] Mehes-Smith M, Nkongolo KK. Physiological and Cytological Responses of *Deschampsia cespitosa* and *Populus tremuloides* to Soil Metal Contamination. Water Air Soil Pollut 2015; 226(4): 1–12.

[17] Kalubi KN, Mehes-Smith M, Narendrula R, Michael P, Omri A. Molecular analysis of red maple (*Acer rubrum*) populations from a reclaimed mining region in Northern Ontario (Canada): soil metal accumulation and translocation in plants. Ecotoxicology. 2015; 24(3): 636–47. 10.1007/s10646-014-1411-7 25560741

[18] Mortazavi A, Williams BA, McCue K, Schaeffer L, Wold B. Mapping and quantifying mammalian transcriptomes by RNA-seq. Nat. Methods 2008; 5: 621–628. 10.1038/nmeth.1226 PMC13303166 18516045

[19] Hossain MZ, Hossain MD, Fujita M. Induction of pumpkin glutathione S-transferases by different stresses and its possible mechanisms. Biol Plantarum. 2006; 50(2): 210–218.

[20] Dixon DP, Edwards R. “Glutathione transferases” The *Arabidopsis* Book. vol. 8 Texas: The American Society of Plant Biologists; 2010; 8:e0131 10.1199/tab.0131 22303257 PMC3244946

[21] Skόrzyńska-Polit E, Drazkiewicz M, Krupa Z. The activity of the antioxidative system in cadmium-treated *Arabidopsis thaliana*. Biol Plantarum. 2004; 47: 71–78.

[22] Gajewska E, Skłodowska M. Differential biochemical responses of wheat shoots and roots to nickel stress: antioxidative reactions and proline accumulation. Plant Growth Regul. 2008; 54: 179–188.

[23] Freeman JL, Persans MW, Nieman K, Albrecht C, Peer W, Pickering IJ, et al Increased Glutathione Biosynthesis Plays a Role in Nickel Tolerance in Thlaspi Nickel Hyperaccumulators. The Plant Cell. 2004; 16(8): 2176–2191. 15269333 10.1105/tpc.104.023036 PMC519206

[24] Jozefczak M, Remans T, Vangronsveld J, Cuypers A. Glutathione Is a Key Player in Metal-Induced Oxidative Stress Defenses. Int J Mol Sci. 2012; 13(3): 3145–3175. 10.3390/ijms13033145 22489146 PMC3317707

[25] Dos Santos CV, Rey P. Plant thioredoxins are key actors in the oxidative stress response. Trends in plant science. 2006; 11(7): 329–334. 16782394 10.1016/j.tplants.2006.05.005

[26] Williams LE, Pittman JK, Hall JL. Emerging mechanisms for heavy metal transport in plants. Biochimica et Biophysica Acta. 2000; 1465: 104–126. 10748249 10.1016/s0005-2736(00)00133-4

[27] Mizuno T, Usui K, Horie K, Nosaka S, Mizuno N, Obata H. Cloning of three ZIP/Nramp transporter genes from a Ni hyperaccumulator plant *Thlaspi japonicum* and their Ni 2+-transport abilities. Plant Physiol Bioch. 2005; 43(8): 793–801. 10.1016/j.plaphy.2005.07.006 16198592

[28] Iannelli MA, Pietrini F, Fiore L, Petrilli L, Massacci A. Antioxidant response to cadmium in *Phragmites australis* plants. Plant Physiol Bioch. 2002; 40(11): 977–982.

[29] Schickler H, Caspi H. Response of Antioxidative Enzymes to Nickel and Cadmium Stress in Hyperaccumulator Plants of Genus *Alyssum*. Physiol Plant. 1999; 105: 39–44.

[30] Gajewska E, Skłodowska M. Effect of nickel on ROS content and antioxidative enzyme activities in wheat leaves. Bio Metals. 2007; 20: 27–36. 10.1007/s10534-006-9011-5 16752220

[31] Yan R, Gao S, Yang W, Cao M, Wang S, Chen F. Nickel toxicity induced antioxidant enzyme and phenylalanine ammonia-lyase activities in Jatropha curcas L. cotyledons. Plant Soil Environ. 2008; 54(7): 294–300.

[32] Pandolfini T, Gabbrielli R, Comparini C. Nickel Toxicity and Peroxidase Activity in Seedlings of *Triticum aestivum* L. Plant Cell Environ. 1992; 15: 719–725.

[33] Seregin V, Kozhevnikova AD. Physiological Role of Nickel and Its Toxic Effects on Higher Plants I. Russ J Plant Physiol. 2006; 53(2): 257–277.

[34] Clemens S. Molecular mechanisms of plant metal tolerance and homeostasis. Planta 2001; 212: 475–486. 11525504 10.1007/s004250000458

[35] Nevo Y, Nelson N. The NRAMP family of metal-ion transporters. BBA-Mol Cell Res. 2006; 1763(7): 609–620. 10.1016/j.bbamcr.2006.05.007 16908340

[36] Kramer U, Talke IN, Hanikenne M. Transition metal transports. FEBS letters. 2007; 581: 2263–2272. 17462635 10.1016/j.febslet.2007.04.010

[37] Sevilla F, Camejo D, Ortiz-Espín A, Calderón A, Lázaro JJ, Jiménez A. The thioredoxin/peroxiredoxin/sulfiredoxin system: current overview on its redox function in plants and regulation by reactive oxygen and nitrogen species. J Exp Bot. 2015: erv146. 10.1093/jxb/erv146 25873657

[38] Lemaire S, Keryer E, Stein M, et al Heavy-Metal Regulation of Thioredoxin Gene Expression in *Chlamydomonas reinhardtii*. Plant Physiol. 1999; 120(3): 773–778. 10398712 10.1104/pp.120.3.773 PMC59315

[39] Wei W, Chai T, Zhang Y, Han L, Xu J, Guan Z. The *Thlaspi caerulescens* NRAMP homologue TcNRAMP3 is capable of divalent cation transport. Mol Biotechnol. 2009; 41(1): 15–21. 10.1007/s12033-008-9088-x 18663607

