# Supplementary figures and images for: Comprehensive Transcriptome Analysis of Response to Nickel Stress in White Birch (Betula papyrifera)
Source: PLoS One. 2016 Apr 15;11(4):e0153762. doi: 10.1371/journal.pone.0153762 (PMC4833294; doi:10.1371/journal.pone.0153762)

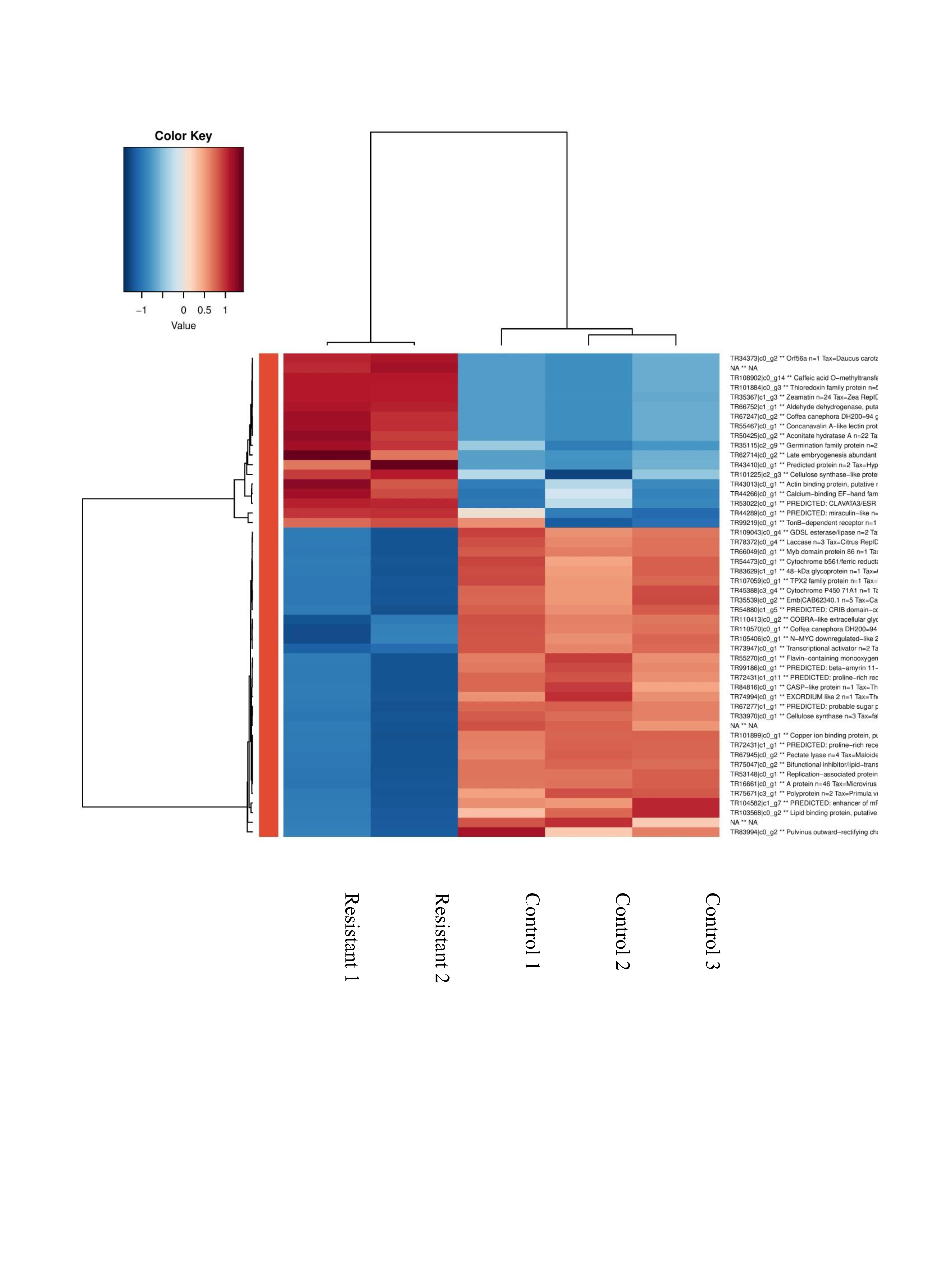

Supplement: S1 Fig — The red colour represents an upregulation and blue downregulation. (TIF) [file pone.0153762.s001.tif]

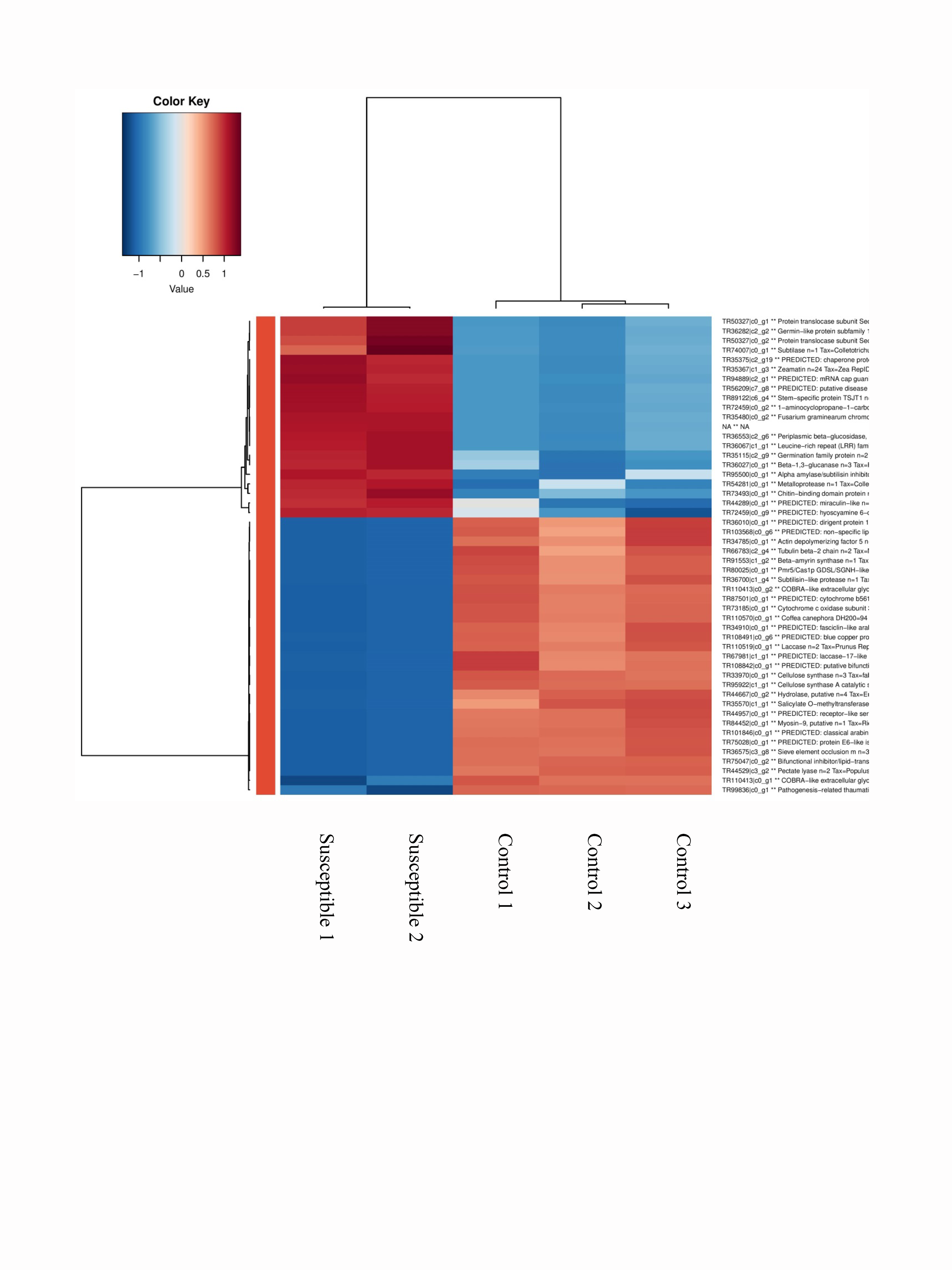

Supplement: S2 Fig — The red colour represents an upregulation and blue downregulation. (TIF) [file pone.0153762.s002.tif]

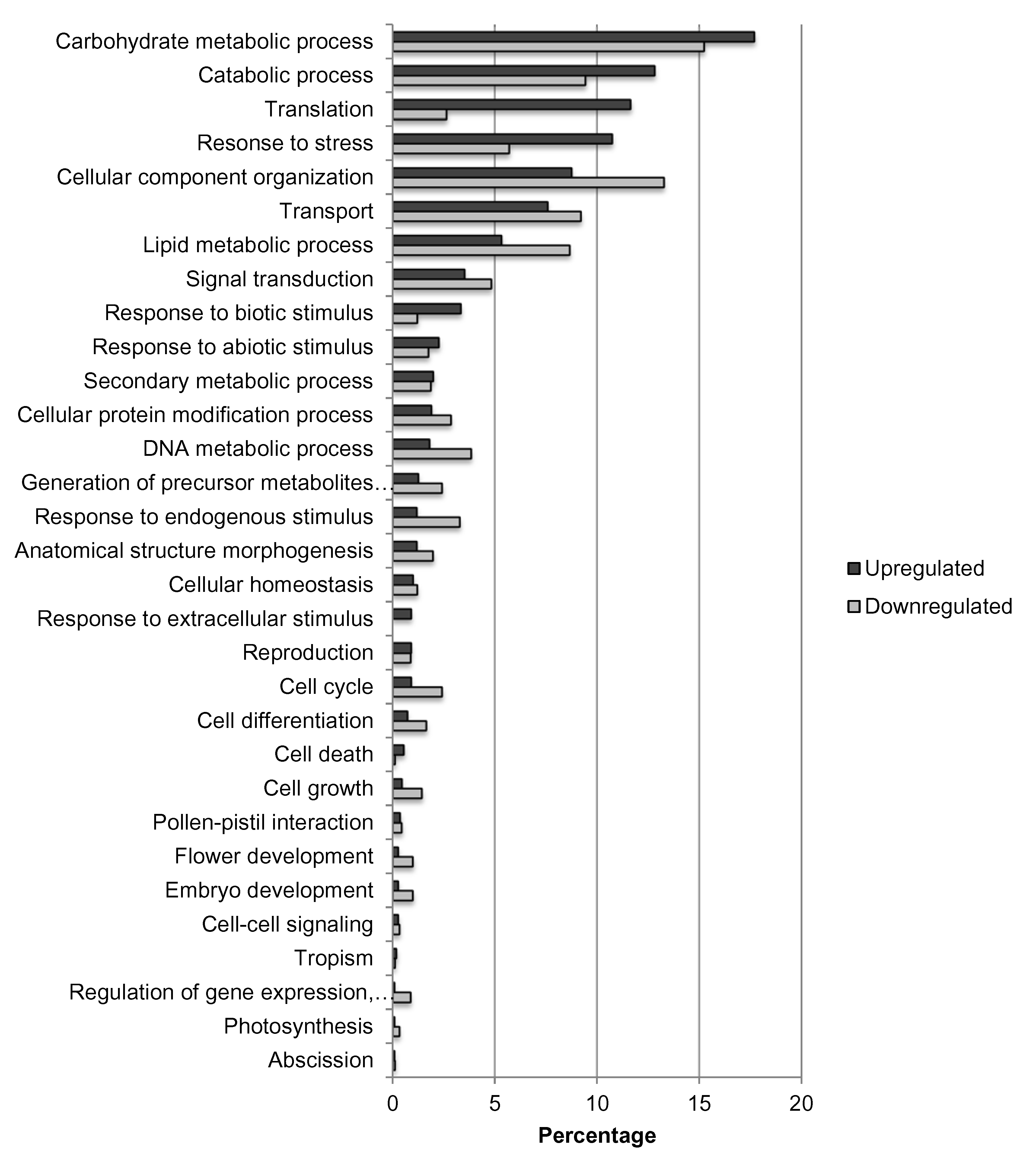

Supplement: S3 Fig — Transcripts were assigned gene ontology and grouped by biological function using BLAST2GO. (TIF) [file pone.0153762.s003.tif]

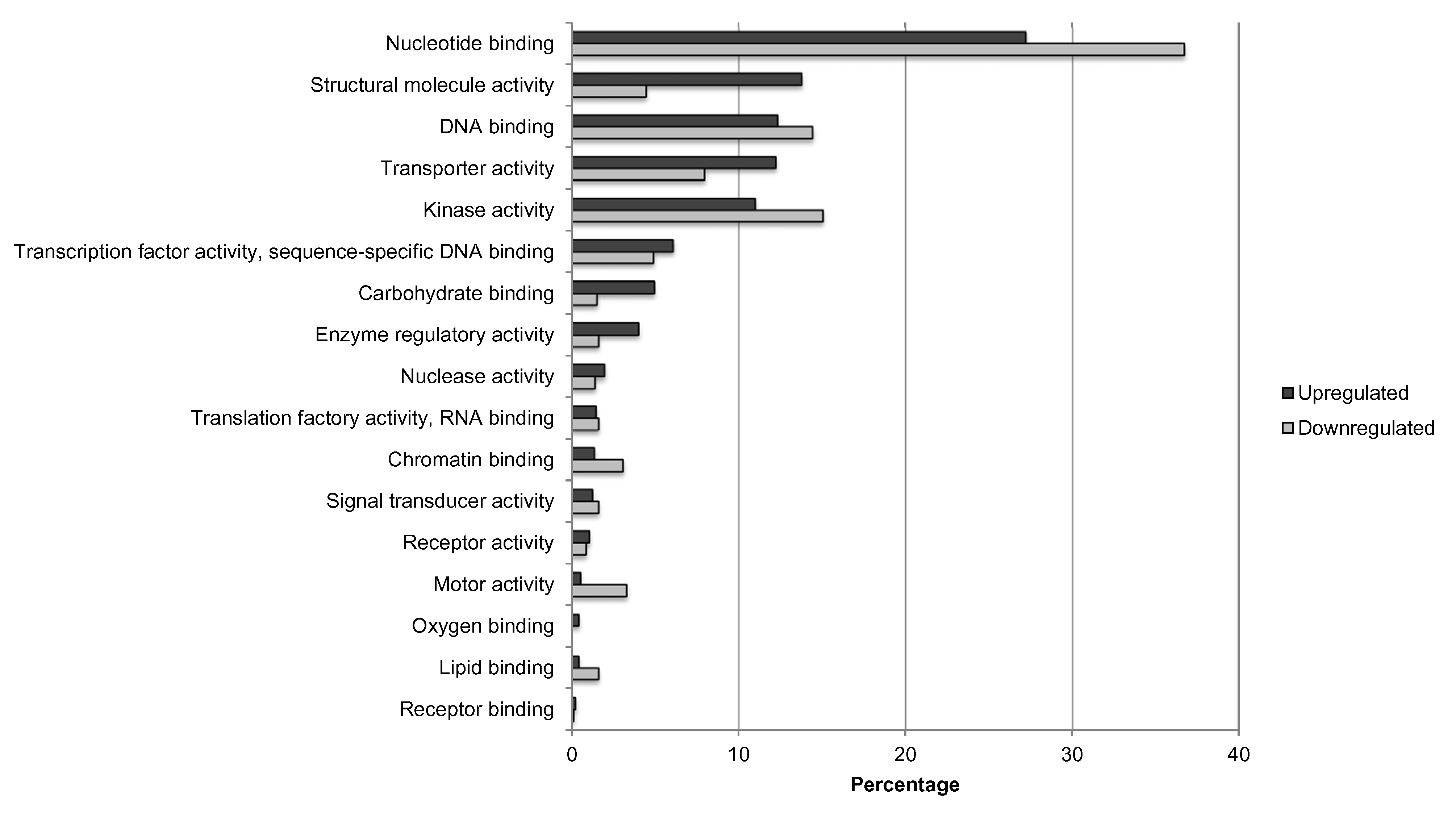

Supplement: S4 Fig — Transcripts were assigned gene ontology and grouped by molecular function using BLAST2GO. (TIF) [file pone.0153762.s004.tif]

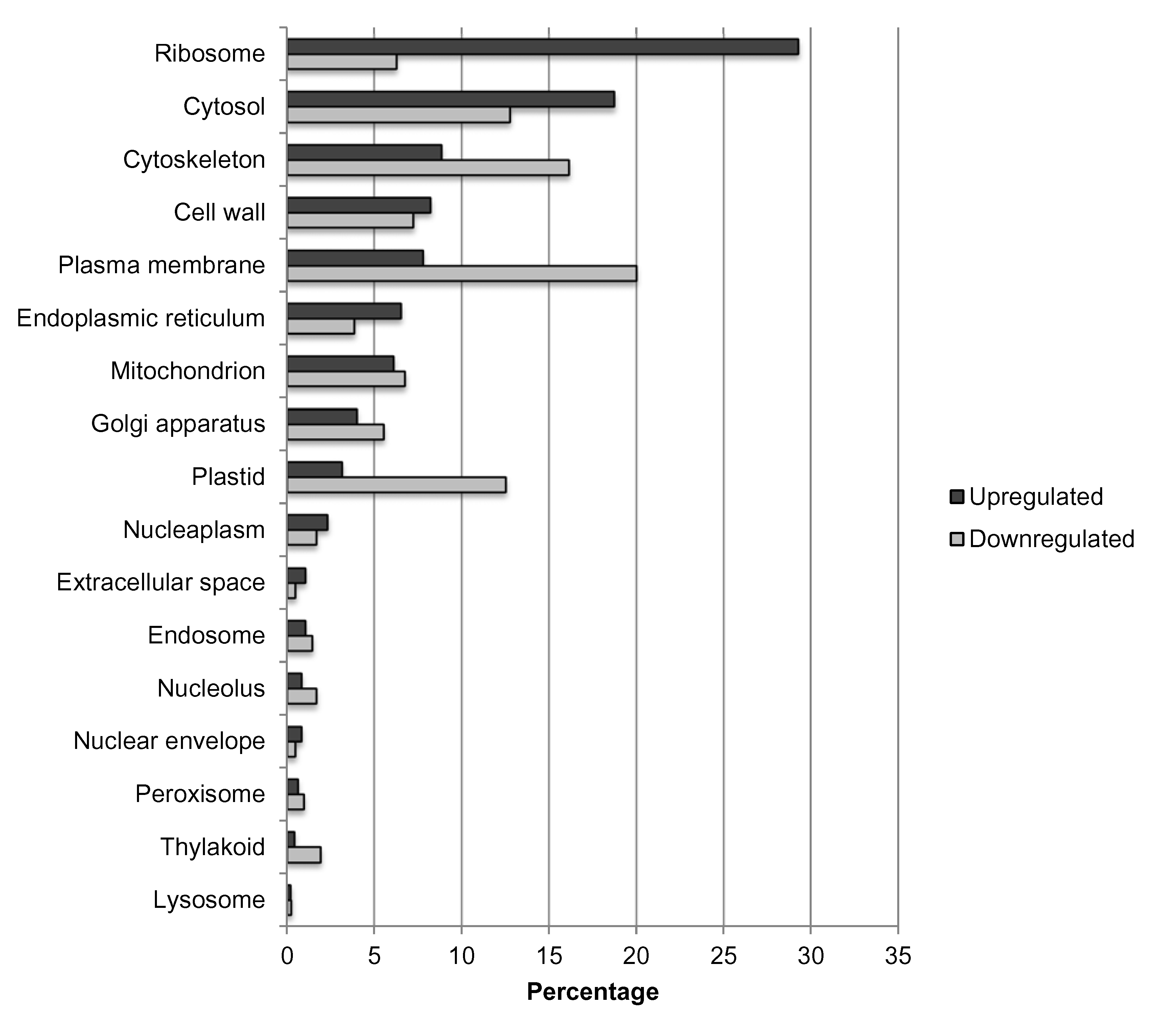

Supplement: S5 Fig — Transcripts were assigned gene ontology and grouped by cellular compartment using BLAST2GO. (TIF) [file pone.0153762.s005.tif]

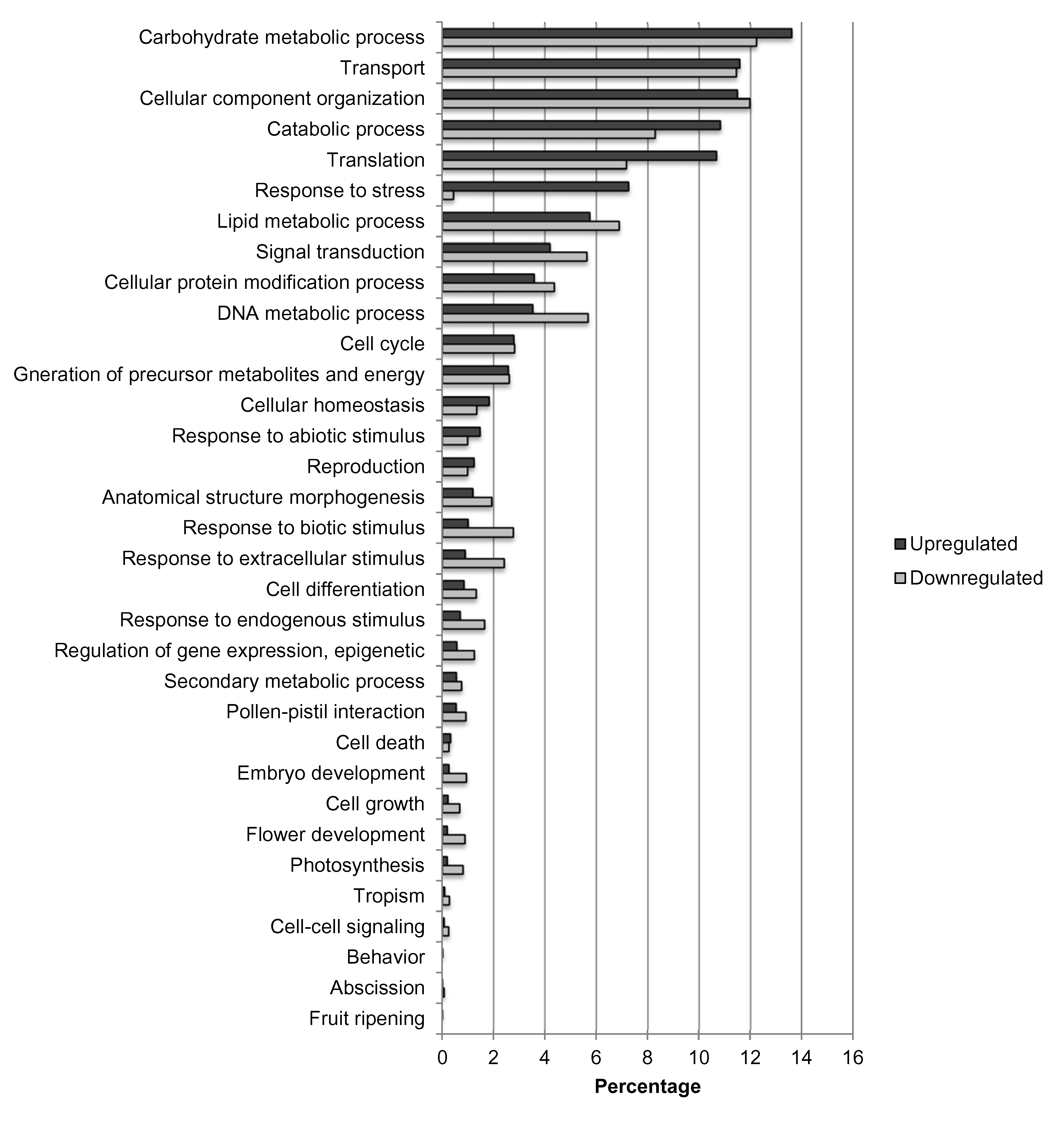

Supplement: S6 Fig — Transcripts were assigned gene ontology and grouped by biological function using BLAST2GO. (TIF) [file pone.0153762.s006.tif]

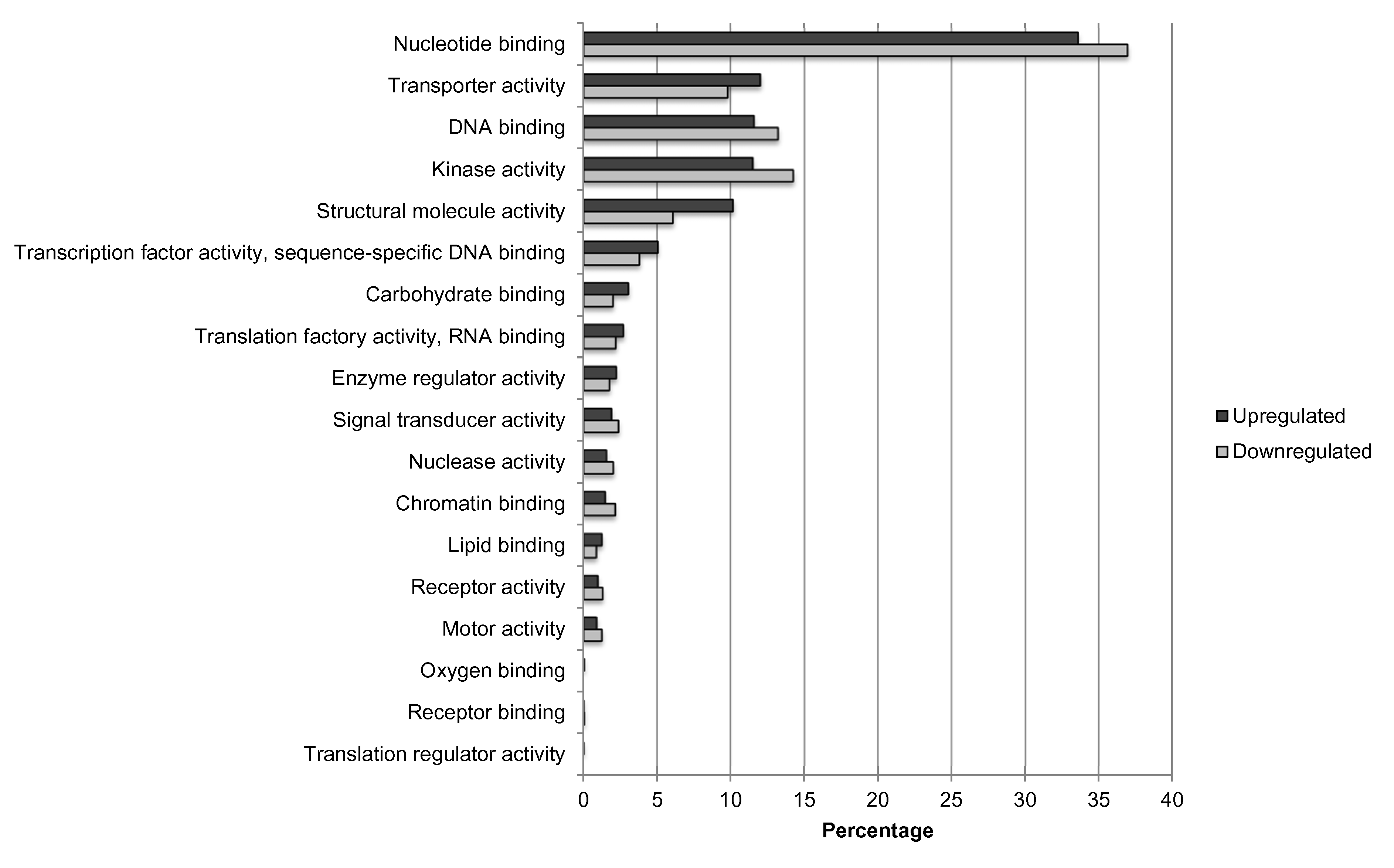

Supplement: S7 Fig — Transcripts were assigned gene ontology and grouped by molecular function using BLAST2GO. (TIF) [file pone.0153762.s007.tif]

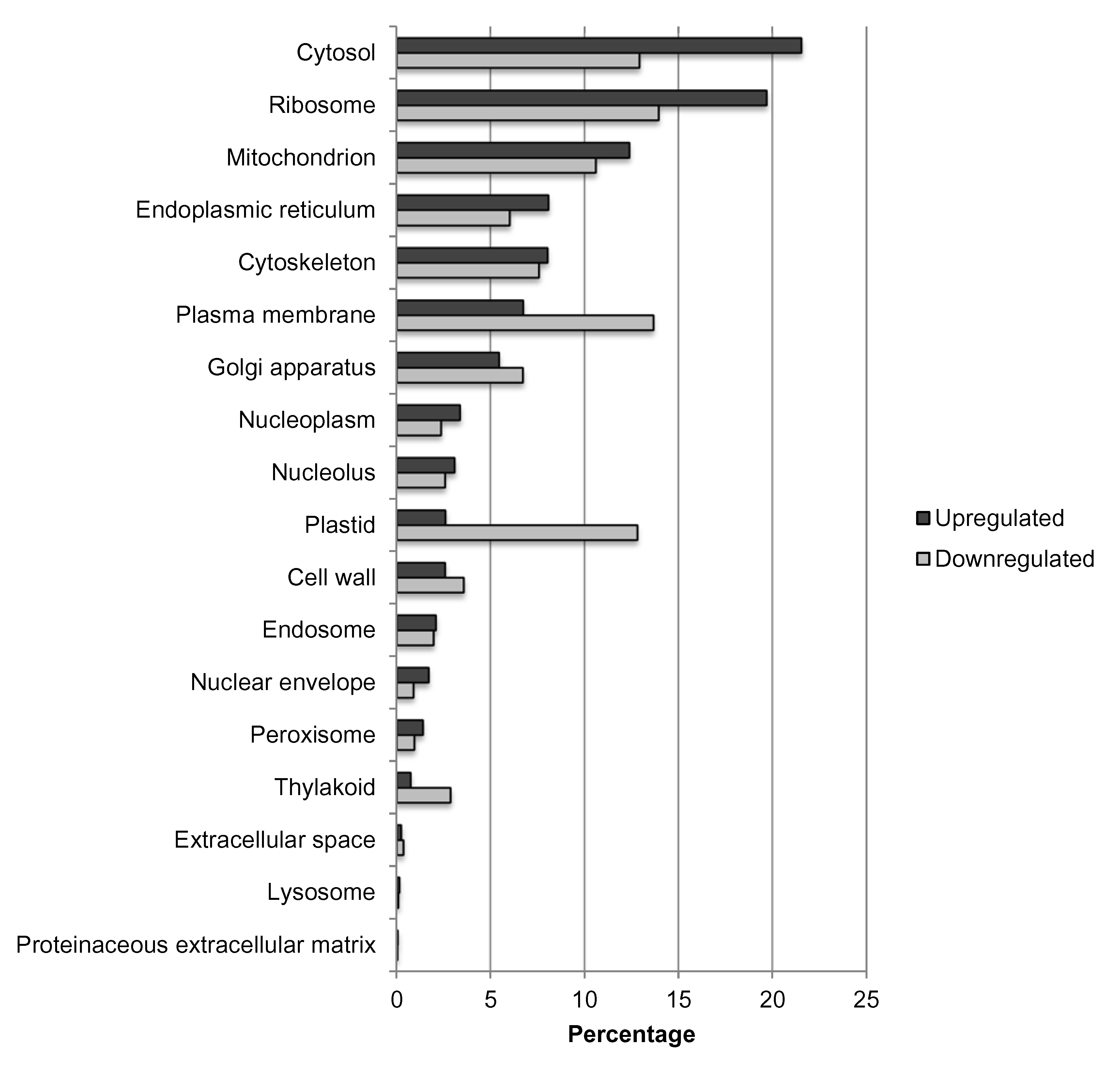

Supplement: S8 Fig — Transcripts were assigned gene ontology and grouped by cellular compartment using BLAST2GO. (TIF) [file pone.0153762.s008.tif]
